# A Strong Tracking Mixed-Degree Cubature Kalman Filter Method and Its Application in a Quadruped Robot

**DOI:** 10.3390/s20082251

**Published:** 2020-04-16

**Authors:** Jikai Liu, Pengfei Wang, Fusheng Zha, Wei Guo, Zhenyu Jiang, Lining Sun

**Affiliations:** 1 State Key Laboratory of Robotics and System, Harbin Institute of Technology, Harbin 150080, China; 12B908035@hit.edu.cn (J.L.); wangpengfei007@163.com (P.W.); wguo01@hit.edu.cn (W.G.); zfsh751228@163.com (L.S.); 2 Shenzhen Academy of Aerospace Technology, Shenzhen 518057, China; sunyu0622@126.com

**Keywords:** quadruped robot, state estimation, STMCKF, IMU, kinematics

## Abstract

The motion state of a quadruped robot in operation changes constantly. Due to the drift caused by the accumulative error, the function of the inertial measurement unit (IMU) will be limited. Even though multi-sensor fusion technology is adopted, the quadruped robot will lose its ability to respond to state changes after a while because the gain tends to be constant. To solve this problem, this paper proposes a strong tracking mixed-degree cubature Kalman filter (STMCKF) method. According to system characteristics of the quadruped robot, this method makes fusion estimation of forward kinematics and IMU track. The combination mode of traditional strong tracking cubature Kalman filter (TSTCKF) and strong tracking is improved through demonstration. A new method for calculating fading factor matrix is proposed, which reduces sampling times from three to one, saving significantly calculation time. At the same time, the state estimation accuracy is improved from the third-degree accuracy of Taylor series expansion to fifth-degree accuracy. The proposed algorithm can automatically switch the working mode according to real-time supervision of the motion state and greatly improve the state estimation performance of quadruped robot system, exhibiting strong robustness and excellent real-time performance. Finally, a comparative study of STMCKF and the extended Kalman filter (EKF) that is commonly used in quadruped robot system is carried out. Results show that the method of STMCKF has high estimation accuracy and reliable ability to cope with sudden changes, without significantly increasing the calculation time, indicating the correctness of the algorithm and its great application value in quadruped robot system.

## 1. Introduction

A legged robot has broad application prospects owing to its strong ability to pass through complex ground without continuous support. To let a quadruped robot gain strong autonomous motion capability, the state estimation of a quadruped robot is a challenge, that is, the robot needs to accurately estimate its state information (position, velocity, attitude) before performing each action, and only in this way can the right decisions be made for the next motion. The inertial measurement unit (IMU) can calculate the state information through the built-in accelerometer and gyroscope, which has been used in almost all mobile robots due to its advantages of good concealment, high short-term accuracy and less susceptibility to environmental interference. However, a long-term use of IMU will cause accumulated error, leading to a drift of calculation results. Some errors are unavoidable in IMU of various levels, which need to be addressed by additional correction measures. Multi-sensor fusion technology offers a solution to this problem. There are mainly two commonly used methods. One is to add additional sensors, such as GPS, camera, which relies on specific external references. However, the information accuracy is prone to environmental interference and the newly added sensors have high requirements for communication and computing equipment, resulting in increased cost and poor reliability. The other method, which does not need additional sensors, is to redevelop the application potential of the system’s own sensors and acquire state information using a forward kinematics solution. This method does not lead to increased cost or accumulated errors, nor require additional communication and computing equipment support, so the implementation is easy. The internal sensor has a low short-term accuracy although it is not affected by the environment. A reasonable idea is to integrate it with IMU to give play to their advantages while abandoning the disadvantages of the two. The fusion effect is mainly determined by a fusion algorithm. Therefore, research on fusion algorithm carries great significance for the development of quadruped robots.

A quadruped robot is basically a non-linear system. The fusion algorithm commonly used in engineering for non-linear systems is extended Kalman filter (EKF) [1]. As it is based on classical Kalman and obtained by solving a Jacobian matrix from an equation of state and observation equation, the principle is simple, easy to understand and easy to realize. EKF also requires calculation of Jacobian matrix, and it is only applicable to weakly nonlinear systems. Moreover, its accuracy is merely equal to first-degree accuracy of Taylor expansion. However, the excellent real-time performance makes it widely used in various non-linear systems including quadruped robots. The unscented Kalman filter (UKF) [2], particle filter (PF) [3] and cubature kalman filter (CKF) [4] represent other types of common non-linear filters with common characteristics. Their common feature is that they all use sampling points to approximately approach non-linear equations. Generally speaking, a larger number of sampling points means better fitness with the non-linear function, and also means higher estimation accuracy. However, the calculation amount of the algorithm is mainly determined by the number of sampling points. Improving accuracy and increasing real-time performance mean a set of contradictory propositions, so these three algorithms have higher accuracy and calculation amount than EKF. There are no uniform UKF sampling strategies and many parameters need to be adjusted manually, so algorithm performance is mainly determined by prior knowledge. PF utilizes a large number of sampling particles in exchange for high estimation accuracy, but its tremendous calculation amount makes it difficult to meet the requirements of online use. CKF is essentially a UKF with a unified sampling strategy, so it has a calculation amount basically comparable to UKF, and can reach third-degree truncation accuracy of Taylor series expansion. Because of its strict mathematical derivation, according to the third-degree spherical diameter volume rule, 2n volume points with the same weight of 1/2n are sampled on the hypersphere surface with variance; for instance, the number is 18 points for a quadruped robot system with a 9-dimensional state equation. It guarantees that the covariance matrix is always a positive definite matrix while avoiding the divergence problem caused by non-positive definiteness of the covariance matrix, so the numerical stability is higher compared to UKF. Gupta D et al. [5] pointed out that CKF has better filtering accuracy than UKF with three-dimensional systems and above. To further improve the accuracy, higher-degree versions such as fifth-degree CKF [6] and seventh-degree CKF [7] have been proposed successively. Although an arbitrary degree CKF is possible in theory, the number of sampling points is required to increase gradually with the continuous improvement of estimation accuracy. The fifth-degree CKF requires $2n^{2}+1$ volume points, and the 9-dimensional system requires 163 volume points. The seventh-degree CKF requires $n^{3}+9n^{2}+14n+6$ volume points, and the 9-dimensional system requires 1590 volume points. It can be seen that although higher degree CKF allows higher estimation accuracy, the number of volume points increases rapidly with the increase of degree. Moreover, CKF of each degree inherits shortcomings of KF and depends more on the initial value. The symmetry and non-negative definiteness of the covariance matrix can sometimes undermine the estimation effect and easily lead to filter divergence. To ensure the symmetry and non-negative definiteness of the covariance matrix and effectively avoid the problem of filter divergence, a square root volume Kalman filter (SRCKF) [8] directly performs recursive updating in the form of square root of the covariance matrix during the filtering process, which can effectively improve numerical stability of filter. To further reduce calculation complexity and achieve higher calculation efficiency, Cui et al. [9] proposed a state of charge (SOC) estimation algorithm based on SRCKF. SRCKF approximates the average of the state variables by calculating 2n points with the same weight according to the cubic transformation. After these points are propagated by the non-linear function, the mean value and variance can reach the third-degree accuracy of the real value of the non-linear function. SRCKF directly propagates and updates the square root of the covariance matrix in the form of Cholesky decomposition, which guarantees the non-negative property of the covariance matrix and avoids the divergence of the filter. Wang et al. [10] proposed SSRCKF by adopting the simplest phase-diameter sampling rule. Compared with the traditional volumetric sampling method, it demonstrates simpler calculation and higher accuracy. Liu et al. [11] proposed a interacting multiple model fifth-degree spherical simplex-radial cubature Kalman filter (IMM5thSSRCKF) by integrating the interacting multiple model (IMM) filter with the fifth-degree spherical simplex-radial cubature Kalman filter (5thSSRCKF). Wang et al. [12] proposed a mixed-degree spherical simple-x-radial cubature Kalman filter (MSSRCKF), and analyzed its accuracy. Simulations results showed that the algorithm can achieve performance improvement of fifth-degree SSRCKF and has higher accuracy under less computational burden. The MSSRCKF collects 2n+3 volume points based on the third-degree surface integrals and fifth-degree radial integrals, which requires only three more volume points than the standard CKF, but achieves an accuracy approaching to the fifth-degree CKF [13]. In the 9-dimensional system, only 21 volume points are required, which saves 142 points for each volume point sampling compared to the fifth-degree CKF. Although it is also reasonable to combine the fifth-degree spherical integral and the third-degree radial integral, $n^{2}+3n+2$ volume points are required, which means, 110 volume points are required in a 9-dimensional system. Hence, MSSRCKF is more suitable for a quadruped robot system.

A quadruped robot is also a time-varying system, whose motion state often changes suddenly. The state equation established based on quadruped robot inertial navigation system is often a highly non-linear high-dimensional time-varying system. To obtain state information accurately in the presence of frequently changed motion state, the filter demands strong robustness and fast convergence speed. Strong tracking [14] is a method that is very suitable for combined use with other filters to improve robust performance of the original filter. Since its inception, it has been incorporated with EKF, known as the strong tracking extended Kalman filter (STEKF) [14]. By introducing fading factor into the state prediction covariance matrix, it adjusts the gain matrix online in real time, and forces the filtering residual sequences to remain mutually orthogonal. In this way, STF can still keep track of the system state when the system model is uncertain, which effectively solves the problems of poor robustness and filtering divergence of EKF under uncertainties. Feng et al. [15] combined strong tracking filtering (STF) [14] with the seventh-degree SSRCKF to obtain higher accuracy. Huang et al. [16] combined strong tracking theory with CKF to solve the problem of spacecraft attitude estimation, but the algorithm demands three times of volume point sampling calculations. Hua et al. [17] proposed the strong tracking spherical simplex-radial CKF (STSSRCKF) algorithm to deal with sudden changes of the target state. However, the shortcoming is that the single fading factor was used and three times of volume point sampling calculations were still required. Zhao et al. [18] proposed the adaptive robust square-root CKF (ARSCKF) algorithm by combining strong tracking theory with SRCKF, However, due to the lack of rigorous derivation and proof to truly satisfy the orthogonality of novelty sequences, it was also necessary to carry out the calculation of cubic volume point sampling. Such traditional strong tracking CKF (TSTCKF) requires three volume point samplings for each filtering [19]. Zhang et al. [19] discussed the calculation method and addition of the location of the fading factor in detail and then proposed the normal strong tracking CKF (NSTCKF) and fast strong tracking CKF (FSTCKF). However the shortcoming is that both algorithms only adopted a single fading factor and the calculation of Jacobian matrix was still required. Although FSTCKF requires only two volume point samplings, compensation is unnecessary for observation prediction. It can be seen that these previous methods combined with strong tracking are basically based on SFEKF combination, and EKF does not need sampling point calculation, but UKF and CKF and some improved algorithms require sampling point calculation, which makes sampling times increase from two to three for each filtering process, which significantly increases the calculation amount and thereby hider the application of quadruped robots.

In order to solve the problem in the above analyses, this paper proposes a strong tracking mixed-degree cubature Kalman filter (STMCKF) and conducts research from the perspective of bionics. According to the structural characteristics of the quadruped robot, the forward kinematics dead reckoning of the robot is obtained in real time through the linear displacement sensor, and the results of the forward kinematics dead reckoning with the IMU are filtered through the fusion of SMSRCKF, and then fusion results are used to correct the IMU. By demonstrating correct combination of strong tracking and CKF-type algorithms, the calculation method of fading factor matrix is improved, and sampling times per filter of TSTCKF is reduced from three to one. Since no additional external sensors are added, external environmental interference is avoided. In this way, the quadruped robot system using this algorithm can significantly improve state estimation accuracy, real-time performance and robustness without increasing research and development costs. The improved STMCKF has fifth-degree accuracy, strong robustness and good real-time performance, which only needs one sampling point calculation.

## 2. Strong Tracking Mixed-Degree Cubature Kalman Filter (STMCKF)

Considering the high complexity of quadruped robot system, it is difficult to establish a system model with sufficient accuracy. In fact, the advantages of a quadruped robot lie in flexibility and strong trafficability because of which quadruped robots often face sudden changes of state. All filters designed with a Kalman filter (KF) as the basic framework, including MSSRCKF, are likely to deteriorate in estimation performance, and even filter divergence may be resulted, which is catastrophic situation for the motion control of quadruped robots. Therefore, it is necessary to adopt strong tracking theory to cope with sudden state changes. The following non-linear time-varying system with additive noise is considered:

(1) $$x_{k}=f_{k-1}\left(x_{k-1}\right)+\mu_{k-1}$$

(2) $$z_{k}=h_{k}\left(x_{k}\right)+\eta_{k}$$

where $x_{k}\in R^{n}$ is state vector of the system at the moment k, $z_{k}\in R^{m}$ is observation vector of the system at the moment k; $f_{k-1}\left(\cdot \right)$ and $h_{k}\left(\cdot \right)$ are, respectively state equation and observation equation of the time-varying system. $\mu_{k-1}\in R^{n}$ is the system process noise, and $\eta_{k}\in R^{m}$ is the system observation noise. They are zero-mean Gaussian white noises that are independent of each other. The variances are $Q_{k-1}$ and $R_{k}$ respectively.

The filtering process of TSTCKF can be described as follows. First, volume point calculation is performed, and then the state prediction ${\hat{x}}_{k/k-1}$ and prediction covariance matrix $P_{k/k-1}$ are calculated; the second volume point calculation is performed, and then the observation covariance matrix $P_{zz,k/k-1}$ and cross covariance matrix $P_{xz,k/k-1}$ are calculated without introducing fading factor. On this basis, the fading factor $\lambda_{k}$ is calculated, and the new $P_{k/k-1}^{*}$ after introduction of the fading factor is calculated. Next, * means introduction of the fading factor.

(3) $$P_{k/k-1}^{*}=\lambda_{k}F_{k-1}P_{k-1}F_{k-1}^{T}+Q_{k-1}$$

where $\lambda_{k}\geq 1$. $F_{k-1}$ is the Jacobian matrix of the state function $f_{k-1}\left(x_{k-1}\right)$ at ${\hat{x}}_{k-1}$; $P_{k-1}$ is the covariance matrix of the state estimate.

Perform the third volume point calculation, then calculate the observation covariance matrix $P_{zz,k/k-1}^{*}$ and cross covariance matrix $P_{xz,k/k-1}^{*}$ after introduction of the fading factor, and then calculate the filtering gain $K_{k}^{*}$, state estimate ${\hat{x}}_{k/k}^{*}$ and estimated covariance matrix $P_{k/k}^{*}$, thus completing all filtering algorithms.

By using the above traditional method, each strong tracking filtering process requires three volume point calculations, so the calculation amount is seriously increased, which is not conducive to practical applications in engineering. For non-linear systems with high dimensionality of state variables or observation variables, this problem is more prominent. In addition, this algorithm not only increases calculation amount, but also destroys optimality of the algorithm. The core of strong tracking filtering theory is that observational innovation meets the principle of orthogonality. The principle of orthogonality means that on the premise of completely accurate system model, the innovation sequence output by Kalman filter is a Gaussian white noise sequence with zero mean, so that these innovation sequences are orthogonal everywhere. To ensure that the algorithm’s optimality and orthogonality principles are both met, the combination of strong tracking and MSSRCKF should meet the following conditions:

(4) $$E[(x_{k}-{\hat{x}}_{k}){\left(x_{k}-{\hat{x}}_{k}\right)}^{T}]=\min$$

(5) $$tr\left\{E[(z_{k+j}-{\hat{z}}_{k+j}){\left(z_{k}-{\hat{z}}_{k}\right)}^{T}]\right\}=tr\left\{E\left[{\tilde{z}}_{k+j}{\tilde{z}}_{k}^{T}\right]\right\}=0$$

where k and j are both positive integers; and ${\tilde{z}}_{k}$ is observational innovation. Formula (4) indicates smallest variance of the filtering; Formula (5) indicates that the residual sequences output by the filters at different times are mutually orthogonal.

Based on the principle of orthogonality, we re-derived the sufficient conditions for the combination of strong tracking and MSSRCKF, and found that the previous combination of STF and non-linear filtering may be incorrect. It is very embarrassing to decide whether to perform compensation for fading factor when calculating the state estimation ${\hat{x}}_{k/k}^{*}$. No matter the innovation ${\tilde{z}}_{k}$ without introduction of fading factor is used, or innovation ${\tilde{z}}_{k}^{*}$ with introduction of fading factor is used, the sufficient conditions for strong tracking filtering to be established cannot be met (see Appendix A for detailed demonstration).

It can be seen from the above analysis that the introduction of fading factor $\lambda_{k}$ in the prediction covariance matrix $P_{k/k-1}$ is obviously inappropriate, which is a suboptimal estimation. According to the standard CKF formula, it can be seen that by introducing the fading factor to the observation covariance matrix $P_{zz,k/k-1}$ and the cross covariance matrix $P_{xz,k/k-1}$, an equivalent strong tracking effect can be obtained. At the same time, it can ensure that the estimation result is unbiased, that is, optimal estimation. Moreover, the correct combination of ST and MSSRCKF can be realized by derivation (see Appendix B for details).

The state equation is a 9-dimensional quadruped robot system. The state error of a single channel requires the same compensation from all other channels, which is obviously not scientific. It is more reasonable to form a fading factor matrix with fading factor to achieve one-to-one correspondence with each state channel and adopt individual compensation for each channel.

The fading factor matrix is designed as follows:

(6) $$\lambda_{k}^{i}=\{\begin{array}{c} \alpha_{i}c_{k},\alpha_{i}c_{k}>1\\ 1,\alpha_{i}c_{k}\leq 1 \end{array},c_{k}=\frac{tr[N_{k}]}{\sum_{i=1}^{n}\alpha_{i}M_{k}^{ii}}$$

where $\alpha_{i}\geq 1$, $\lambda_{k}^{i}$ is the element of the fading factor matrix; the calculation of $N_{k}$ and $M_{k}$ is shown in Appendix B. $M_{k}^{ii}$ is the element on the diagonal of the matrix $M_{k}$; $\alpha_{i}$ value is obtained by RBF neural network training, which can also be obtained by prior knowledge. Multiple fading factors can adjust different state variables with different fading speeds, which fully reflects the dynamic change of internal state of the entire filter and improves adaptive ability of the filter. When $\alpha_{i}=1$, the fading factor matrix is equivalent to a single fading factor.

It can be seen that fading factor plays a role of adaptive adjustment according to the changes in the residual, which avoids the majority of calculation process involving volume point, thus greatly reducing the calculation burden.

It can be seen that when the system state does not change suddenly or the system model is accurate, the above formula is established.

(7) $$E[{\tilde{z}}_{k}{\tilde{z}}_{k}{}^{T}]=V_{k}=H_{k}P_{k/k-1}H_{k}^{T}+R_{k}$$

However, when the state changes suddenly or the model is inaccurate, the innovation will inevitably increase, so that orthogonality is no longer met. That is, formula (4) is not established. At the same time, $V_{k}$ increases, the filtering result is no longer optimal.

(8) $$V_{k}>H_{k}P_{k/k-1}H_{k}^{T}+R_{k}$$

To ensure filtering optimality, the innovation sequence is forced to be orthogonal, so as to extract as much valid information as possible from the measurement innovation. Therefore, if a single fading factor $\lambda_{k}\geq 1$ is introduced into the variance matrix $P_{k/k-1}$ of state prediction error, then

(9) $$V_{0,k}=H_{k}(\lambda_{k}\left(P_{k/k-1}-Q_{k-1}\right)+Q_{k-1})H_{k}^{T}+R_{k}$$

It can be seen that when the model is accurate or there is no sudden change in the state, that is $\lambda_{k}=1$, strong tracking filter becomes invalid and degenerates to the original filter. 

For the sake of reducing computation, a determination of whether strong tracing is needed. The strong tracking filter should play a role when needed, and strong tracking function is unnecessary when there is no sudden system change. In this way, a filtering convergence criterion is proposed herein: if the system state changes suddenly, the actual estimation error is usually larger than the prediction error, and the error covariance matrix can be approximately considered as unbounded. This modeling indicates quadratic sum of the innovation sequence ${\tilde{z}}_{k}^{T}{\tilde{z}}_{k}$ includes the actual estimation error. According to this feature, filtering convergence criteria is constructed based on the nature of the innovation sequence.

(10) $${\tilde{z}}_{k}^{T}{\tilde{z}}_{k}>\alpha tr\left\{H_{k}P_{k/k-1}H_{k}^{T}+R_{k}\right\}$$

where formula (10) is a criterion for judging whether the filter is converged or not. α≥1 means adjustable coefficient. If the formula (10) is established, it means that the actual filtering error exceeds the theoretical prediction error by ε times, and the filter diverges. At this time, strong tracking is required to give full play to its observation role to suppress the filter divergence. On the contrary, if the formula (10) is not established, it means that the filter works normally. At this stage, it is possible to estimate the state variable with MSSRCKF alone without strong tracking.

STMCKF is described in detail below. It is specifically stated that there are two versions of this method, and the following version is universal. STMCKF in Section 3 is a simplified version to reduce calculation amount, which is suitable for systems that can linearize the observation equation. Since the main research object of this paper is the quadruped robot, the simulation and experiment are carried out for the quadruped robot, and other systems will not be discussed. 

### 2.1. Initialization 

Assume that state estimation ${\hat{x}}_{k-1}$ and state variance matrix $P_{k-1}$ at the moment k−1 are known, take a set of vectors $a_{i}={\left[a_{i,1},a_{i,2},\ldots ,a_{i,n}\right]}^{T},i=1,2,\ldots ,n+1$, where, n is the state dimension.

(11) $$a_{i,j}=\{\begin{array}{c} -{\left[\frac{n+1}{n(n-j+2)(n-j+1)}\right]}^{0.5},j<i\\ {\left[\frac{\left(n+1)(n-i+1\right)}{n(n-i+2)}\right]}^{0.5},j=i\\ 0,j>i \end{array}$$

Volume point of the filter is indicated as follows:

(12) $$\{\begin{array}{c} \xi_{i}=0\;,i=0\\ \xi_{i}={\left[\sqrt{\left(n+2\right)}a\right]}_{i}\;,i=1,2,\ldots ,n+1\\ \xi_{i}=-{\left[\sqrt{\left(n+2\right)}a\right]}_{i}\;,i=n+2,\ldots ,2n+2 \end{array}$$

where ${\left[\;\right]}_{i}$ means n -dimension point set obtained by calculation, the number of points is n+1, and the weight corresponding to the volume point is:

(13) $$\{\begin{array}{c} \omega_{i}=\frac{2}{n+2}\;,i=0\\ \omega_{i}=\frac{n}{2(n+1)(n+2)}\;,i=1,2,\ldots ,2n+2 \end{array}$$

### 2.2. Predication 

We need calculate $\xi_{i}$ according to formulas (11) and (12); singular value decomposition (SVD) is a decomposition method applicable to any matrix. It has a computational complexity equivalent to Cholesky decomposition, but avoids error in the filtering process caused by non-positive definiteness of mean square error matrix in one-step prediction after multiple cycles, thus improving numerical calculation robustness while enhancing filtering accuracy. As a result, SVD is adopted for the calculation of volume points.

SVD of the state variance $P_{k-1}$ at the moment k−1 is performed:

(14) $$P_{k-1}=U_{k-1}S_{k-1}V_{k-1}^{T}$$

Calculate the first volume point $X_{i,k-1},i=0,1,\ldots ,2n+2$:

(15) $$X_{i,k-1}={\hat{x}}_{k-1}+U_{k-1}\sqrt{S_{k-1}}\xi_{i},i=0,1,\ldots ,2n+2$$

where $S_{k-1}=diag\left(s_{1,}s_{2},\ldots ,s_{r},0,\ldots ,0\right)\;,s_{1}>s_{2}>\ldots >s_{r}>0$ is the singular value of the matrix $P_{k-1}$; $U_{k-1},S_{k-1},V_{k-1}\in R^{n\times n}$.

The first volume point spread:

(16) $$\chi_{i,k/k-1}=f_{k-1}\left(\chi_{i,k-1}\right)\;,i=0,1,\ldots ,2n+2$$

Calculate weight $\omega_{i}$ of each volume point according to formula (13), and then calculate the one-step predicted value of the state:

(17) $${\hat{x}}_{k/k-1}=\sum_{i=0}^{2n+2}\omega_{i}\chi_{i,k/k-1}\;,i=0,1,\ldots ,2n+2$$

Calculate one-step prediction covariance matrix of the state:

(18) $$P_{k/k-1}=\sum_{i=0}^{2n+2}\omega_{i}\chi_{i,k/k-1}\chi_{i,k/k-1}^{T}-{\hat{x}}_{k/k-1}{\hat{x}}_{k/k-1}^{T}+Q_{k-1}$$

If the observation equation is a non-linear function, then a second volume point sampling is needed and fading factor matrix should be calculated.

### 2.3. Pseudo Observation Update

Since the observation equation is changed into the pseudo observation equation, the linear equation *H_k_* satisfies the following equations:

(19) $${\hat{z}}_{k/k-1}=H_{k}{\hat{x}}_{k/k-1}$$

(20) $$P_{xz,k/k-1}=P_{k/k-1}H_{k}^{T}$$

### 2.4. State Mutation Test

When a sudden change in the robot’s state is detected, STMCKF initiates strong tracking mode. After improvement, the number of sampling point calculation in TSTCKF can be reduced from three to one, and a new multiple fading factor matrix is given, thus achieving unbiased estimation; when there is no sudden state change, STMCKF initiates normal mode without adopting strong tracking. Use formula (10) to check whether strong tracking is needed.

### 2.5. Calculate Multiple Fading Factors 

Calculate the fading factor matrix according to formula (6).

### 2.6. Recalculation 

Recalculate one-step prediction variance matrix $P_{k/k-1}^{*}$ and the new root mean square $S_{k/k-1}^{*}$ after adding the fading factor. ${}^{*}$ means after adding the fading factor.

(21) $$P_{zz,k/k-1}^{*}=\lambda_{k}\left(1:m,1:m\right)M_{k}+R_{k}$$

(22) $$P_{xz,k/k-1}^{*}=\lambda_{k}P_{xz,k/k-1}$$

In the formula, since the modified observation equation has m*n dimensional matrix, the fading factor matrix takes the square matrix composed of the previous m dimensions to perform channel compensation.

### 2.7. Update

The third volume point sampling is no longer needed, and the state estimate and variance are directly calculated:

(23) $$K_{k}^{*}=P_{xz,k/k-1}^{*}/P_{zz,k/k-1}^{*}$$

(24) $${\hat{x}}_{k}^{*}={\hat{x}}_{k/k-1}+K_{k}^{*}\left(z_{k}-{\hat{z}}_{k/k-1}\right)$$

(25) $$P_{k}^{*}=\lambda_{k}P_{k/k-1}-K_{k}^{*}P_{xz,k/k-1}^{*\;T}$$

For systems where equation of state is non-linear, Traditional Strong Tracking CKF (TSTCKF) requires three volume point calculations for each filtering, but STMCKF only needs one volume point calculation for each filtering.

## 3. Forward Kinematics of Quadruped Robot

Years of research work on mammals and arthropods has shown that the animal’s own movement and position can be effectively estimated based on its own inertial information and joint information. For a quadruped robot, a link coordinate system is established according to its leg structure. By calculating forward kinematics of the robot, motion parameters of the robot can be obtained simply and conveniently.

The IMU used in this work is a NAV440CA-200 developed by MEMSIC (MEMSIC, Wuxi, China), which is a cheap civilian IMU, costing about $1500. To improve its accuracy and suppress the drift, this paper adopts linear displacement sensors of each joint of the quadruped robot to calculate the joint angle, and calculates the displacement of each foot relative to the body through positive kinematics so as to obtain the position and velocity of the legged robot and provide redundant calibration information for the IMU. The IMU itself has very accurate pitch angle and roll angle. It is difficult to obtain yaw angle through the combination navigation of positive kinematics and IMU, and other auxiliary sensors are needed for correction. 

**Figure 1:**
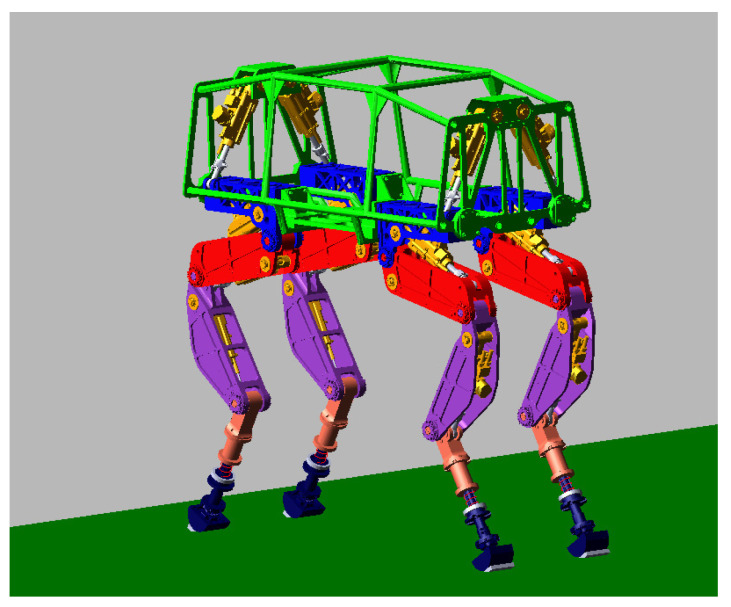
Three-dimensional (3D) model of the quadruped robot.

The three-dimensional model of the quadruped robot studied in his paper is shown in Figure 1. Each leg of the robot includes five joints: side swing, forward side-sway hip joint, front hip joint, knee-joint, ankle joint and passive line elastic component. Each active joint is driven by a hydraulic cylinder and is equipped with a displacement sensor.

**Figure 2:**
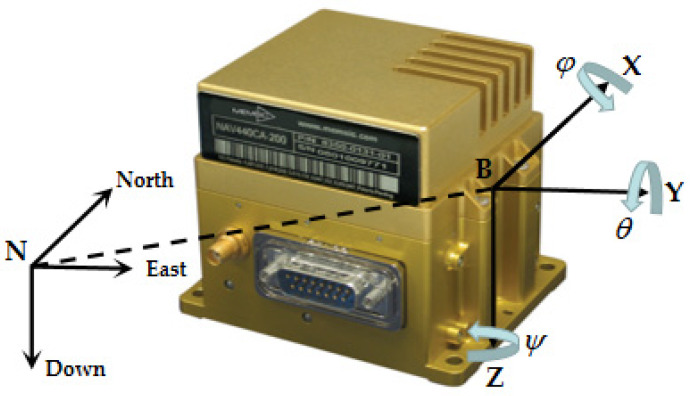
The navigation coordinate system N and the body coordinate system B.

Figure 2 shows the definition of the navigation coordinate system N and the body coordinate system B. It is worth noting that IMU placement position has been accurately fixed at the center of mass of the quadruped robot in the design phase. Without consideration of external load design, the lever arm error is ignored here.

The direction cosine matrix $C_{b}^{n}$ from N to B can be calculated according to the yaw angle ψ around the Z axis, the pitch angle θ around the Y axis and the roll angle φ around the X axis, as shown in Equation (26).

(26) $$\begin{array}{ll} C_{b}^{n} & =C(\psi )C(\theta )C(\varphi )\\ & \begin{array}{c} =\left[\begin{array}{ccc} \cos\psi & -\sin\psi & 0\\ \sin\psi & \cos\psi & 0\\ 0 & 0 & 1 \end{array}\right] \end{array}\left[\begin{array}{ccc} \cos\theta & 0 & \sin\theta \\ 0 & 1 & 0\\ -\sin\theta & 0 & \cos\theta \end{array}\right]\left[\begin{array}{ccc} 1 & 0 & 0\\ 0 & \cos\varphi & -\sin\varphi \\ 0 & \sin\varphi & \cos\varphi \end{array}\right]\\ & \begin{array}{c} =\left[\begin{array}{ccc} \cos\psi \cos\theta & l & m\\ \sin\psi \cos\theta & n & q\\ -\sin\theta & \cos\theta \sin\varphi & \cos\theta \cos\varphi \end{array}\right] \end{array} \end{array}$$

(27) l=−sinψcosφ+cosψsinθsinφ

(28) m=sinψsinφ+cosψsinθcosφ

(29) n=cosψcosφ+sinψsinθsinφ

(30) q=−cosψsinφ+sinψsinθcosφ

**Figure 3:**
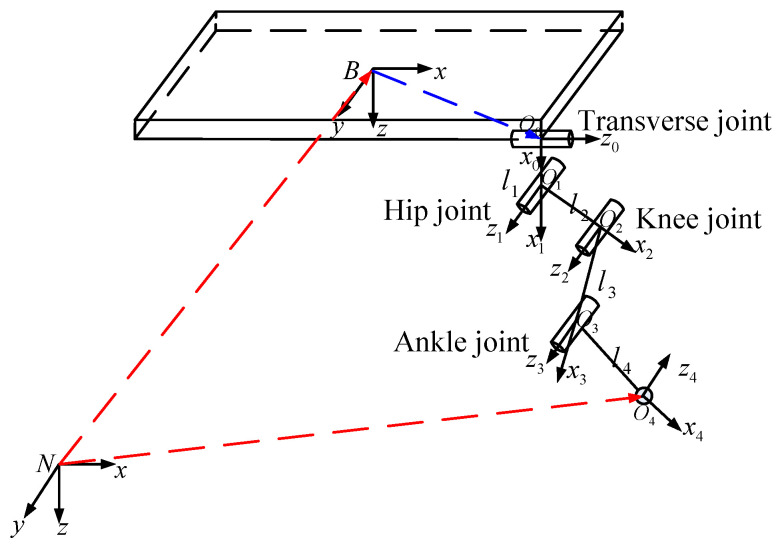
Connecting rod coordinate system of right front leg of quadruped robot [20].

The quadruped robot adopts ankle joint opposite vertex design, and its four legs are symmetrically arranged, so forward kinematic analysis is made only with the right front leg support ground as an example. Since the displacement of the passive linear elastic link is small and not easy to measure, it has little influence on the kinematics calculation. Therefore, the robot’s leg linkage coordinate system is ignored. According to the establishment principle of DH joint coordinate system, the coordinate system of the link from the robot center of mass to the foot end is obtained, as shown in Figure 3. According to the joint link coordinate system, a D-H parameter table from side hip joint to the foot end is established, as shown in Table 1.

Where the connecting rod angles are transverse joint angle $\theta_{1}$, hip joint angle $\theta_{2}$, knee joint angle $\theta_{3}$ and ankle joint angle $\theta_{4}$, which can be calculated in accordance with the leg structure and hydraulic cylinder configuration. By calculating the homogeneous transform matrix $A_{10}\sim A_{43}$ between adjacent joints, the homogeneous transform matrix $A_{0B}$ (front hip joint coordinate system relative to body coordinate system), and the homogeneous transform matrix $A_{BN}$ (body coordinate system relative to navigation coordinate system), as shown in the equation. 

The homogeneous transform matrix $A_{4N}$ (foot coordinate system relative to navigation coordinate system) can be obtained, as shown in Equation (31).

(31) $$A_{4N}=A_{BN}A_{0B}A_{10}A_{21}A_{32}A_{43}$$

In calculation of kinematic velocity of mass center, the arithmetic product of joint angular velocity and the Jacobian matrix is avoided. In the moving process of a quadruped robot, serious shocks will occur, and spaces will exist in leg joints, causing errors of displacement sensor data. Therefore, directly using differential to calculate angular velocity will enlarge calculation errors.

The specific method is to calculate robot body displacement distance for every 10 sampling periods (sampling frequency is over 200 Hz). The average velocity can be gained, as shown in Equation (32). This method can remove the impact of shocking vibration and effectively improve the calculation accuracy. Considering the real-time performance, 0.05 s of lag is within the permissible range.

(32) $$\bar{v}=\frac{\Delta s}{10T}$$

where *T* is the sampling periods. $\bar{v}$ is the average locomotion velocity within 10 sampling periods. Δs is the robot body displacement within 10 sampling periods.

### 3.1. Equation of State for Quadruped Robot 

Forward kinematics of the quadruped robot is used to correct the position and velocity innovation of the IMU, which can make full use of high response rate and high accuracy of the IMU. Moreover, long-term drift of the IMU can be corrected via positive kinematics solution. The IMU has very accurate pitch and roll angles. When the quadruped robot is walking in dynamic gait, that is, when it is landing on one or two feet, it is impossible to determine its attitude angle only using positive kinematics. We need to estimate the position and velocity of the quadruped robot. To dynamically compensate the deviation of the accelerometer, the deviation values of the accelerometers on the three axes in the IMU are extended to the state variables of the filter. In this way, 9 state variables are needed. Here, it is worth noting that, the north direction of the IMU is consistent with the robot’s forward direction, the east direction of the IMU points horizontally to the right side of the robot, and the vertically downward direction of the IMU points to the bottom of the robot.

To realize dynamic bias compensation, strapdown inertial navigation system (SINS) bias as state variable is introduced into the state equation in this work. The outputs of accelerator and gyroscope include bias and noise. As shown in Equation (33), the derivative of bias and random error is regarded as the Gaussian noise.

(33) $$\begin{array}{ll} a=\hat{a}-b_{a}-w_{a} & w_{a}\sim N\left(0,Q_{a}\right)\\ {\dot{b}}_{a}=w_{ba} & w_{ba}\sim N\left(0,Q_{ba}\right) \end{array}$$

where $Q_{a},Q_{ba}$ represent the variance of random errors, a is the real value of the accelerator, $\hat{a}$ is the measured value of the accelerator, $b_{a}$ is the bias of accelerator, $w_{a}$ is the random error of the accelerator and $w_{ba}$ is the random error of accelerator bias.

The differential equation of the state variable should be established before deriving the state equation. The velocity updating differential equation of SINS is expressed as Equation (34):

(34) $$\overset{\cdot }{v}=C_{b}^{n}a-\left(2\omega_{e}^{n}+\omega \right)v+g^{n}$$

where v is the robot velocity in navigation coordinate system, $\omega_{e}^{n}$ is the projection of earth rotational angular velocity in navigation coordinate system, ω is the projection of angular velocity of body coordinate relatively to earth coordinate in navigation coordinate system. $g^{n}$ represents the acceleration of gravity in the navigation coordinate system.

In Equation (34), $\left(2\omega_{e}^{n}+\omega \right)v$ represents the Coriolis acceleration term and centripetal acceleration term, which can be regarded as harmful acceleration during velocity calculation. For mobile robot, the moving velocity is normally less than 2 m/s, the order of magnitude for $\omega_{e}^{n}$, ω is only 10^−4^. It can be known that the magnitude of coriolis acceleration is 10^−5^ and the magnitude of centripetal acceleration is only 10^−7^, which is negligible in velocity calculation. The velocity upgrading differential equation of SINS can be simplified as Equation (35). Subsequently, the integral operation is performed using the Runge–Kutta method, so that the real time velocity variation can be obtained.

(35) $$\overset{\cdot }{v}\approx C_{b}^{n}a+g^{n}$$

where $C_{b}^{n}a+g^{n}$ is the accelerometer measurement value, which can also be written as $C_{b}^{n}\left(\hat{a}-b_{a}-w_{a}\right)+g^{n}$. As $C_{b}^{n}$ is time-varying, the discrete recursive form of each state component can be written as follows:

(36) $$s_{k}=s_{k-1}+v_{k-1}t+\frac{t^{2}}{2}\left(C_{b,k-1}^{n}\left({\hat{a}}_{k-1}-b_{a,k-1}-w_{a,k-1}\right)+g^{n}\right)$$

(37) $$v_{k}=v_{k-1}+t\left(C_{b,k-1}^{n}\left({\hat{a}}_{k-1}-b_{a,k-1}-w_{a,k-1}\right)+g^{n}\right)$$

(38) $$b_{a,k}=b_{a,k-1}+w_{ba,k-1}$$

where $s_{k}\in R^{3\times 1}$ represents the position of the robot in the navigation coordinate system at the moment k; $v_{k}\in R^{3\times 1}$ represents the speed of the robot in the navigation coordinate system at the moment k; $b_{a,k}\in R^{3\times 1}$ represents the deviation of the accelerometer in the navigation coordinate system at the moment k; t is the sampling time, $C_{b,k-1}^{n}$ is the direction cosine matrix at the moment k−1.

It is written in matrix form as:

(39) $$\left[\begin{array}{c} s_{k}\\ v_{k}\\ b_{a,k} \end{array}\right]=\left[\begin{array}{ccc} I & t & -\frac{t^{2}}{2}C_{b,k-1}^{n}\\ 0 & I & -tC_{b,k-1}^{n}\\ 0 & 0 & I \end{array}\right]\left[\begin{array}{c} s_{k-1}\\ v_{k-1}\\ b_{a,k-1} \end{array}\right]+\left[\begin{array}{c} \frac{t^{2}}{2}\left(C_{b,k-1}^{n}{\hat{a}}_{k-1}+g^{n}\right)\\ t\left(C_{b,k-1}^{n}{\hat{a}}_{k-1}+g^{n}\right)\\ 0 \end{array}\right]+\left[\begin{array}{c} -\frac{t^{2}}{2}C_{b,k-1}^{n}w_{a,k-1}\\ -tC_{b,k-1}^{n}w_{a,k-1}\\ w_{ba,k-1} \end{array}\right]$$

However, the magnitudes of these parameters differ significantly from each other. The error values of displacement, velocity, accelerator bias are regarded as state variables, which can be expressed by Equation (40):

(40) $$x_{k}={\left[\begin{array}{ccc} \Delta s_{k} & \Delta v_{k} & \Delta b_{a,k} \end{array}\right]}^{T}$$

where $\Delta s_{k}$ is the error value of displacement at the moment k, $\Delta v_{k}$ is the error value of velocity at the moment k, $\Delta b_{a,k}$ is the error value of accelerator bias at the moment k.

Take the error values of position, speed and deviation as state variables, then:

(41) $$\left[\begin{array}{c} \Delta s_{k}\\ \Delta v_{k}\\ \Delta b_{a,k} \end{array}\right]=\left[\begin{array}{ccc} I & t & -\frac{t^{2}}{2}C_{b,k-1}^{n}\\ 0 & I & -tC_{b,k-1}^{n}\\ 0 & 0 & I \end{array}\right]\left[\begin{array}{c} \Delta s_{k-1}\\ \Delta v_{k-1}\\ \Delta b_{a,k-1} \end{array}\right]+\left[\begin{array}{c} -\frac{t^{2}}{2}C_{b,k-1}^{n}\Delta w_{a,k-1}\\ -tC_{b,k-1}^{n}\Delta w_{a,k-1}\\ \Delta w_{ba,k-1} \end{array}\right]$$

So the equation of state (41) can be rewritten as:

(42) $$x_{k}=F_{k-1}x_{k-1}+\mu_{k-1}$$

(43) $$F_{k-1}=\left[\begin{array}{ccc} I & t & -\frac{t^{2}}{2}C_{b,k-1}^{n}\\ 0 & I & -tC_{b,k-1}^{n}\\ 0 & 0 & I \end{array}\right]$$

(44) $$\mu_{k-1}=\left[\begin{array}{c} w_{s,k-1}\\ w_{v,k-1}\\ w_{ba,k-1} \end{array}\right]=\left[\begin{array}{c} -\frac{t^{2}}{2}C_{b,k-1}^{n}\Delta w_{a,k-1}\\ -tC_{b,k-1}^{n}\Delta w_{a,k-1}\\ \Delta w_{ba,k-1} \end{array}\right]=\left[\begin{array}{cc} -\frac{t^{2}}{2}C_{b,k-1}^{n} & 0\\ -tC_{b,k-1}^{n} & 0\\ 0 & I \end{array}\right]\left[\begin{array}{c} \Delta w_{a,k-1}\\ \Delta w_{ba,k-1} \end{array}\right]$$

If the variance of $\Delta w_{a,k-1}$ is $Q_{a,k-1}$, the variance of $\Delta w_{ba,k-1}$ is $Q_{b,k-1}$, the process noise variance is:

(45) $$Q_{k-1}=E\left(\Delta w_{a,k-1}\Delta w_{a,k-1}^{T}\right)=\left[\begin{array}{ccc} \frac{t^{4}}{4}C_{b,k-1}^{n}C_{b,k-1}^{n\;T}Q_{a,k-1} & \frac{t^{3}}{2}C_{b,k-1}^{n}C_{b,k-1}^{n\;T}Q_{a,k-1} & 0\\ \frac{t^{3}}{2}C_{b,k-1}^{n}C_{b,k-1}^{n\;T}Q_{a,k-1} & t^{2}C_{b,k-1}^{n}C_{b,k-1}^{n\;T}Q_{a,k-1} & 0\\ 0 & 0 & Q_{b,k-1} \end{array}\right]$$

### 3.2. Pseudo-Observation Equation of Quadruped Robot

Previously, the displacement $s_{f}$ and moving velocity $v_{f}$ can be obtained according to robot forward kinematics analysis, here the two parameters are adopted as measurement variables, based on which the measurement model and the other two parameters (the displacement after correction $s_{c}$ and the velocity after correction $v_{c}$ can be established shown as Equation (46) and simply denoted as Equation (47). 

For the quadruped robot system, the observation equation is linearized. Based on this, STMCKF is proposed. Only one volume point calculation is needed for each filtering, which greatly reduces the calculation amount and improves real-time performance.

(46) $$z_{k}=\left[\begin{array}{c} s_{f}-s_{c}\\ v_{f}-s_{c} \end{array}\right]=\left[\begin{array}{c} \Delta s_{k}\\ \Delta v_{k} \end{array}\right]+\eta_{k}=\left[\begin{array}{ccc} I & 0 & 0\\ 0 & I & 0 \end{array}\right]\left[\begin{array}{c} \Delta s_{k}\\ \Delta v_{k}\\ \Delta b_{a,k} \end{array}\right]+\eta_{k}$$

where $\eta_{s,k}$ and $\eta_{v,k}$ are uncorrelated white Gaussian noises of variance $R_{s}$ and $R_{v}$, respectively. The above equation can be written in the form of Equation (47).

(47) $$z_{k}=Hx_{k}+\eta_{k}$$

For the quadruped robot system herein, $H\in R^{6\times 9}$, the observation noise covariance is:

(48) $$R_{k}=E\left(\eta_{k}\eta_{k}^{T}\right)=diag\left(R_{s},R_{v}\right)$$

### 3.3. Multi-Sensor Fusion Structure Diagram

**Figure 4:**
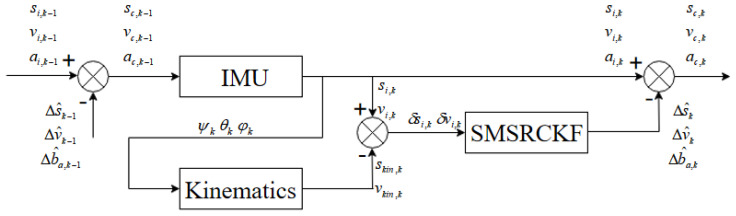
Multi-sensor fusion structure diagram.

The multi-sensor fusion structure diagram is as shown in Figure 4:

(49) $$\left[\begin{array}{c} s_{i.k}\\ v_{i,k} \end{array}\right]=\left[\begin{array}{c} s_{c,k-1}\\ v_{c,k-1} \end{array}\right]+\left[\begin{array}{c} tv_{c,k-1}+\frac{t^{2}}{2}a_{c,k-1}\\ ta_{c,k-1} \end{array}\right]=\left[\begin{array}{c} s_{c,k-1}\\ v_{c,k-1} \end{array}\right]+\left[\begin{array}{c} tv_{c,k-1}+\frac{t^{2}}{2}\left[C_{b,k-1}^{n}\left({\hat{a}}_{k-1}-\Delta b_{a,k-1}\right)+g^{n}\right]\\ t\left[C_{b,k-1}^{n}\left({\hat{a}}_{k-1}-\Delta b_{a,k-1}\right)+g^{n}\right] \end{array}\right]$$

(50) $$\left[\begin{array}{c} s_{c,k}\\ v_{c,k}\\ a_{c,k} \end{array}\right]=\left[\begin{array}{c} s_{i,k}\\ v_{i,k}\\ a_{i,k} \end{array}\right]-\left[\begin{array}{c} \Delta {\hat{s}}_{k}\\ \Delta {\hat{v}}_{k}\\ C_{b,k}^{n}\Delta {\hat{b}}_{a,k} \end{array}\right]$$

where $s_{i,k}$, $v_{i,k}$ and $a_{i,k}$ are navigation calculation results at the moment k. $\Delta \;{\hat{s}}_{k}$, $\Delta \;{\hat{v}}_{k}$ and $\Delta {\hat{b}}_{a,k}$ are estimations of displacement and velocity errors at the moment k. $s_{c,k}$, $v_{c,k}$ and $a_{c,k}$ are position, velocity, and acceleration results of the robot in the navigation coordinate system after correction at the moment k.

The velocity $v_{b,k}$ of the robot body coordinate system may be needed for control. It can be obtained according to formula (51).

(51) $$v_{b,k}=\left[\begin{array}{ccc} \cos\left(\psi \right) & \sin\left(\psi \right) & 0\\ -\sin\left(\psi \right) & \cos\left(\psi \right) & 0\\ 0 & 0 & 1 \end{array}\right]v_{c,k}$$

## 4. Numerical Experiments 

**Figure 5:**
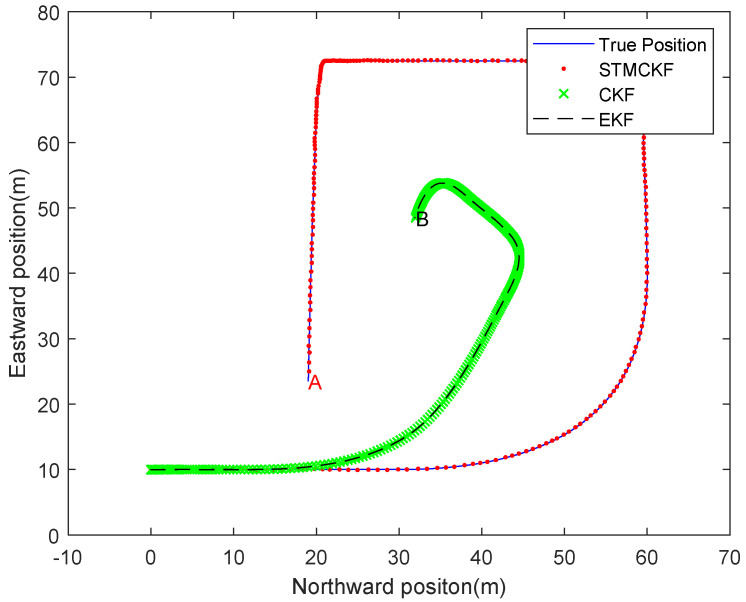
Moving trajectory of quadruped robot in one Monte Carlo run.

The state equation (42) and observation equation (47) are simulated. The sampling time is set to t=0.05s, Given biased parameters $Q_{a,0}$ and $Q_{b,0}$ are eye (3), $R_{k}$ is eye (6), the initial state is $x_{0}=[0;\;10;\;0;\;0;\;0;\;0;\;0;\;0;\;0]$, and $P_{0}$ is eye (9). In the simulation, the actual process noise $\mu_{k}$ and the observation noise $\eta_{k}$ are uncorrelated white Gaussian noise, the average value of $\mu_{k}$ is [0.0659; 0.0703; 0.0033; −0.2846; 0.1933; 0.1704; 0.1901; −0.1339; 0.2395], the variance of $\mu_{k}$ is [9.8559; 8.1359; 8.5398; 9.4093; 10.1551; 7.9678; 7.9462; 9.1310; 9.2863]⋅eye (9); the average value of $\eta_{k}$ is [0.0042; 0.0034; −0.0043; 0.0044; 0.0068; 0.0042], the variance of $\eta_{k}$ is [0.0096; 0.0094; 0.0105; 0.0098; 0.0096; 0.0103]⋅eye (6). The root mean square error (RMSE) is used as the comparison criterion in the simulation. Suppose that the number of simulation times is 300 and the number of Monte Carlo simulation times is 50. The North-East moving trajectory of quadruped robot by Monte Carlo simulation is shown in Figure 5.

**Figure 6:**
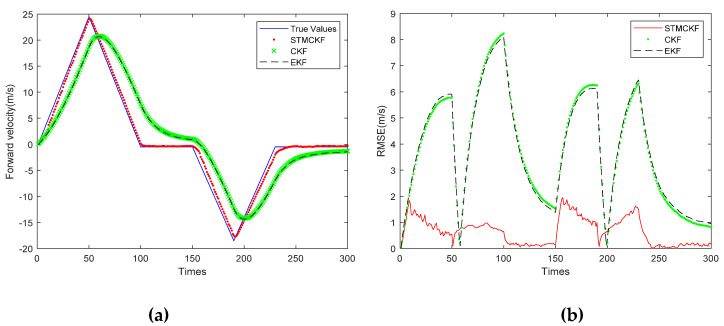
Comparison of forward velocity estimation with three filters. (**a**) Forward velocity estimation; (**b**) RMSE of forward velocity.

In the North-East moving trajectory of quadruped robot simulated by Monte Carlo, the solid line represents the real position moving track, the red dot line represents the position moving track estimated by STMCKF, the green cross line represents the position moving track estimated by CKF, and the black dotted line represents the position moving track estimated by EKF. In order to test the ability of the algorithm to deal with the sudden change of the motion state, some extreme conditions are set artificially in the simulation. From the starting point, the robot accelerates uniformly in the first 50 samples, and then decelerates and turns eastward. After that, it accelerates and moves eastward for a certain distance before decelerating to zero and turning southward. Subsequently, it accelerates again and turns westward for an emergency stop before accelerating again. Finally it decelerates gradually to point A. It can be seen that the estimated position trajectory of STMCKF is very close to the real position trajectory, which verifies the correctness and effectiveness of STMCKF. In the process of the robot suddenly changing motion states at high speed, STMCKF converges rapidly, with only a few sampling periods and a high tracking accuracy maintained, which indicates that STMCKF has a strong robustness. In the stable acceleration stage, the position estimation of CKF and EKF is accurate, but the estimated trajectory starts to deviate from the real trajectory seriously when the robot turns from the lateral acceleration. Finally, the robot is located at point B, which is far from the real position. There are two main reasons for this result: one is the poor robustness of CKF and EKF, and second is the process noise of the quadruped robot system, which leads to the performance degradation of CKF and EKF estimation. It can be seen from the figure that the curves of CKF and EKF are very close, which is mainly due to the nonlinear degree of the state equation of the system is low and EKF has equal estimation ability as CKF. The estimation accuracy of EKF is lower than that of CKF when the degree of weak non-linearity is enhanced. The red full line represents the velocity estimation, the short blue dash line represents the forward kinematics calculation value, the black dot dash line represents the results of navigation velocity calculation, and the long green dashed line represents the real motion parameter. It can be seen from the figure that due to the existence of deviation, a fast drift occurs in velocity calculation. Under the impact of linear displacement sensor noise, the error of forward kinematics calculation is relatively large. However, after data fusion the velocity estimation is very close to its real value. Analytic calculation of error between estimation of velocity value and real velocity value is as follows: 

Figure 6b shows the RMSE of the robot forward velocity estimated by these three filters. In Figure 6a, the solid line represents the true value, the dotted line represents the estimated value of STMCKF, the cross line represents the estimated value of CKF, the dotted line represents the estimated value of EKF, the horizontal axis represents the number of the sampling, the sampling interval is 0.05 s, and the vertical axis represents the forward velocity. It can be observed that the peak forward velocity reaches 25 m/s. This study simulates the harsh working conditions of high-speed motion, large change of acceleration and long sampling interval. Such large sampling intervals present a great challenge to state estimation for systems whose states change frequently. It can be seen from Figure 6a that the estimated curve of STMCKF tracks the real value well. Although the curve lags behind due to the influence of harsh working conditions, the overall estimated result is relatively accurate. The estimation curves of CKF and EKF are similar, indicating that the system is a weak non-linear system and has little impact on the estimation performance of EKF. However, both of them are affected by bad working conditions, and the estimation curves lag significantly. Nevertheless, we can see that the general trend is close to the real value, which shows the correctness of the two. By comparing the estimation curves of three kinds of filters, the superiority and robustness of STMCKF can be proved preliminarily. From Figure 6b, it can be seen that the RMSE of the solid line representing STMCKF is far lower than that of the circle point representing CKF and that of the dotted line representing EKF, indicating that the estimation accuracy of STMCKF is much higher than the other two. From the RMSE curves of CKF and EKF, it can be seen that each sudden change of the robot’s motion state makes RMSE increase rapidly, and the larger the robot’s speed is, the larger the RMSE is, and the smaller the robot’s speed is, the smaller the RMSE is. The RMSE curve of STMCKF can be divided into six sections of 0~50, 50~100, 100~150, 150~190, 190~230, and 230~300, which is completely consistent with the range of motion state change in the simulation, indicating that STMCKF can accurately identify the change of motion state. From the sampling interval of 0~50 and 150~190, it can be seen that the RMSE of STMCKF increases slightly upon the sudden change of the motion state, and does not increase continuously like CKF and EKF. The acceleration of the robot is increasing, but the RMSE of STMCKF shows an obvious downward trend, which shows that STMCKF can recognize in a timely way the change of the motion state, start strong tracking to resist so as to improve its own robustness. It can be clearly seen that 50 and 190 are turning points for emergency stop and turning, while CKF and EKF have a lag of about 10 sampling cycles. The forward speed of the robot decreases rapidly and then turns to an increase. This process can be seen from the RMSE curve of CKF and EKF, but not seen so obviously from the RMSE curve of STMCKF, indicating that STMCKF has a certain inhibition effect on the adverse effects of the sudden change of the robot’s motion state. From the range of 100~150 and 230~300, it can be seen that STMCKF has higher estimation accuracy when the robot’s motion state changes slowly, which is in line with our expectation. This proves the correctness of the simulation experiment and shows the superiority of STMCKF compared with CKF and EKF.

**Figure 7:**
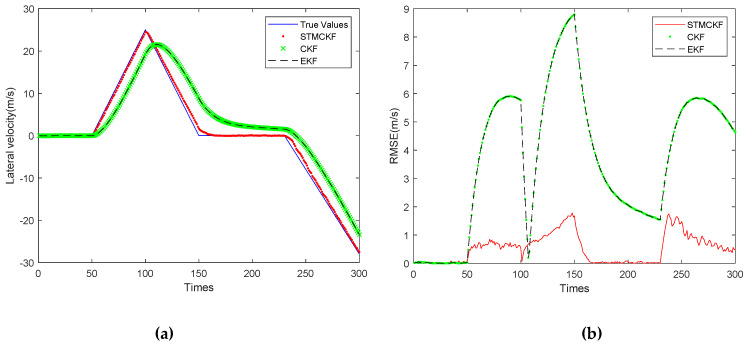
Comparison of lateral velocity estimation with three filters. (**a**) Lateral velocity estimation; (**b**) RMSE of lateral velocity.

Figure 7a shows the lateral velocity estimation curves of the three filters. Figure 7b shows the RMSE curve of the robot’s lateral velocity estimated by three filters. As shown in Figure 7a, the solid line represents the true value, the dotted line represents the estimated value of STMCKF, the cross line represents the estimated value of CKF, the dashed line represents the estimated value of EKF, the horizontal coordinate represents the sampling times, the sampling interval is 0.05 s, and the vertical coordinate represents the forward velocity. It can be seen that the peak lateral velocity reaches −27.2 m/s, which is due to the simulation of high-speed movement of the body and large change in acceleration. This extreme situation has exceeded the range of motion ability of foot-type robot. In addition, the sampling interval is long, which poses a great challenge to state estimation of the system with a drastic change of motion state. It can be seen from Figure 7a that the estimated curve of STMCKF tracks the real value well. The estimation error is obvious at the 150th and 232nd sampling, and there is a slight lag in the whole curve, which is mainly due to the large interval sampling and is difficult to overcome completely. It can also be said that the estimated curve is close to the real value on the whole and the estimated result is accurate. The estimation curves of CKF and EKF are similar, indicating that the system belongs to a weak non-linear system. In this system, the estimation performance of EKF and CKF has little difference, but both of them are affected by bad working conditions, and the estimation curve lags significantly. However, it can be seen that the overall trend is close to the real value, which indicates the correctness of the two filters in the simulation. By comparing the estimation curves of three kinds of filters, the superiority and robustness of STMCKF can be preliminarily proved. From Figure 7b, it can be seen that in the first 50 sampling periods, all three filters have high accuracy, this is because the robot only has forward acceleration and the lateral velocity is almost 0 during this period. The solid line representing the STMCKF shows the lateral acceleration applied at 50, 100, and 230, and the RMSE of the three STMCKF increases slightly in response to the sudden change of motion state, which does not diverge as far as CKF and EKF. STMCKF can suppress the divergence in a few sampling periods, showing excellent robustness.

The running time of EKF, CKF, STMCKF is comparatively studied using a laptop with an Intel Core i5-9400F processor and 8GB RAM, as shown in Table 2.

## 5. Velocity Estimation Experiment of Quadruped Robot 

**Figure 8:**
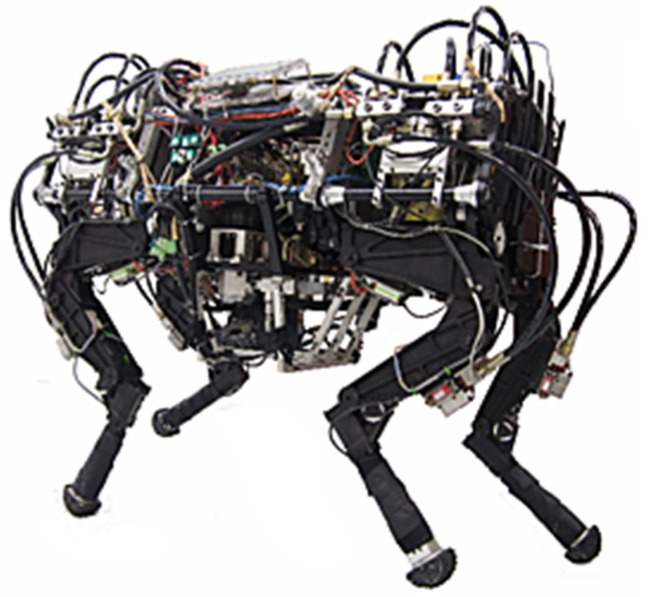
Platform of quadruped robot.

The experimental prototype of the quadruped robot employed is shown in Figure 8. The robot body is equipped with a power system consisting of engine, variable pump, and hydraulic accessories, providing power for the hydraulic cylinder of each joint. 

**Figure 9:**
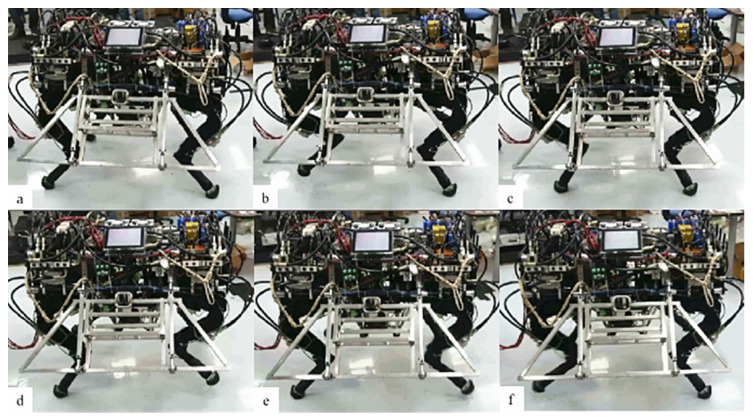
Screenshot of walking experiment of quadruped robot prototype. (**a**) Robot is ready to start a new trot gait cycle; (**b**) Robot raises the left front leg and the right hind leg on the diagonal; (**c**) Robot chooses landing points according to state estimation result; (**d**) Robot raises the right front leg and the left hind leg on the diagonal; (**e**) Robot chooses landing points according to update state estimation result; (**f**) Robot starts next cycle of trot gait.

During the experiment, the quadruped robot was walking on the flat ground in a trot gait at a speed of 1 m/s. The sampling frequency of the IMU is 100 HZ, and the sampling frequency of linear displacement sensor is 200 HZ. Let t=0.005s, $Q_{a,k}=eye\left(3\right)$, $Q_{b,k}=1.25e^{-3}\cdot eye\left(3\right)$, $Q_{k}$ is calculated according to equation (41) based on the value of $C_{b,k}^{n}$; Generally, we use the Suge–Husa method for identification, but in order to make the comparison experiment more convincing, we use the time-invariant observation noise variance in the experiment [0.01; 0.01; 0.01; 0.0001; 0.005; 0.005]⋅eye (6). In the walking process of the robot, STMCKF is used to provide the north and east position of the robot for positioning and navigation, and the forward velocity and lateral velocity of the robot are provided for motion control. Figure 9 is a video screenshot of a quadruped robot walking with STMCKF. It can be seen from the figure that when a pair of diagonal legs are in stable supporting posture, the robot mass center will move forward and the whole process is stable. The correctness and validity of the STMCKF proposed in this paper are proved again by experiments.

**Figure 10:**
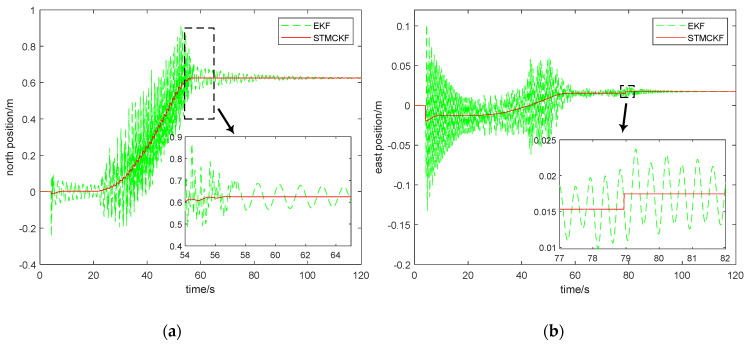

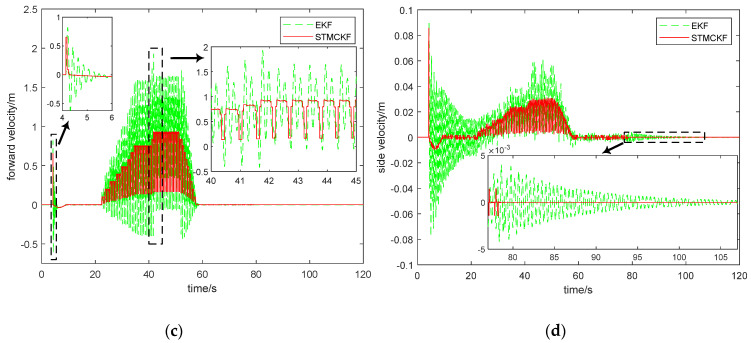
Comparison of state estimation between EKF and STMCKF of a quadruped robot. (**a**) Comparison of north position estimation between EKF and STMCKF; (**b**) Comparison of east position estimation between EKF and STMCKF; (**c**) Comparison of forward velocity estimation between EKF and STMCKF; (**d**) Comparison of forward velocity estimation between EKF and STMCKF.

STMCKF is compared with traditional EKF to highlight the estimation effect of STMCKF more intuitively. For the sake of ensuring the accuracy and fairness of the comparison, we record the data of multiple groups of IMU, and apply STMCKF and EKF to estimate the states of the same group of IMU data, respectively. Here, a 2-minute IMU data is selected, and the comparison diagram of state estimation between STMCKF and EKF for this period of data is shown in Figure 10.

In Figure 10, the red full line represents STMCKF estimation, and green dash line represents EKF estimation. According to Figure 10, it can be seen that it takes about 4 s for the robot to start up and complete initialization, which is consistent with the actual situation. After resetting each foot, the sudden change of the position and velocity of the robot body in all directions will occur, STMCKF and EKF both respond to the sudden change of the robot’s motion state, indicating high sensitivity of the two. However, from the estimation curve, it can be seen that STMCKF converges more rapidly than EKF, and the response amplitude of STMCKF is significantly smaller than EKF, indicating that STMCKF has a strong resistance effect to sudden change of motion state. These results prove that STMCKF is better than EKF in coping with the sudden change of motion state. The robot moves forward at 21 s. In Figure 10a,b, the time-varying curves of the robot’s north and east positions related to the robot’s positioning and navigation performance are depicted. It can be seen that the solid line representing STMCKF presents a regular change with time, indicating that the STMCKF estimation results are accurate. The state switching during 21 s~22 s and 56 s~58 s is smooth and fast, which shows that STMCKF has a strong tracking ability to the motion state and the application of STMCKF can effectively improve the accuracy and stability of positioning and navigation. As shown in Figure 10a,b, the dotted line representing EKF responds to the sudden change at 4s and shows a trend of convergence although it does not fully converge until 21 s. As the robot moves forward, the original convergence trend of EKF becomes the divergence trend and then the robot moves rhythmically, so that the motion state will constantly change and the divergence trend of EKF will converge no more. According to the local enlarged figure in Figure 10a, the robot does not stop moving until 57 s, and STMCKF accurately estimates the static state of the robot and converges directly to the final north position. Although EKF converges gradually from the vibration due to the robot’s static state, it will not converge to the exact final position eventually. The information informs the robot “you are still moving back and forth”, which is obviously unfavorable for positioning and navigation. The partial enlarged view in Figure 10b shows a slight change of the robot’s east position near 79 s, with a displacement of 2.2 mm. Since the moving range of the robot can be ignored, so no one noticed it during the experiment. By comparing Figure 10c with Figure 10d, it can be seen that the robot stops walking after 57 s. The forward velocity of the robot returns to zero, but the lateral speed changes within a very small range. That is to say, the robot swings slightly independently to maintain balance. Instead of swinging back and forth, the robot swings laterally to maintain the balance, which is in line with our bionic design. Since the robot cannot completely calm down after walking, the change of its velocity is notified to the robot control center via STMCKF. Finally, the control center decides to shift 2.2 mm eastward to keep balance. It can be seen from Figure 10d that after the robot moves 2.2 mm eastward, its lateral velocity basically returns to zero. This shows the role of STMCKF in adjusting the motion state of the robot. If EKF is used to transfer the results to the control center, it is difficult for the control center to solve the balance problem via a slight shift because the dotted line in the local enlarged view in Figure 10b,d is constantly oscillating. This shows the advantage of STMCKF in control efficiency. Figure 10c shows that STMCKF converges rapidly in 0.05 s after responding to the sudden change of forward speed caused by robot start-up, while EKF converges successfully in 2 s. EKF is not good at changing the acceleration of the robot. When the walking velocity of the robot is accelerating from zero to the expected velocity of 1 m/s, the estimated results of EKF show a obvious divergent trend, and the amplitude is very large. The estimation velocity of EKF is much higher than the expected velocity, with negative velocity even occurring, which is obviously incorrect. Furthermore, it can be seen from the local enlarged figure of 40 s~45 s that the curve of EKF’s forward velocity estimation is sharp, indicating the velocity often changes abruptly, which is very unfavorable for the control of the quadruped robot. Using such a state estimator will make the track of the foot end of the robot become less smooth, increasing the wear of various components and reducing the service life. The velocity estimation curve plotted by STMCKF shows a regular periodic sawtooth wave, and the peak value of velocity is very close to 1 m/s. This may be because the ground on which the robot walks is made of smooth tile, so the slipping phenomenon occurred, which reduced the walking efficiency.

## 6. Conclusions

To further increase the accuracy of state estimation for a quadruped robot system, a strong tracking mixed-degree cubature Kalman filter algorithm is proposed in this paper. The proposed algorithm is used to fuse IMU and forward kinematics for estimation, which can greatly improve the accuracy, robustness and real-timeness of state estimation without increasing the cost. This algorithm improves the robustness of STMCKF by strong tracking. Moreover, the more appropriate combination of STMCKF and strong tracking is demonstrated in this study. Unlike the traditional combination method of a strong tracking cubature Kalman filter, this paper puts forward a new calculation method of fading factor matrix, which realizes the unbiased estimation and reduces the original calculation of sampling points from three times to once. Through the judgment of the sudden change of motion state, it can be known that the strong tracking can only be started when necessary, which further improves the real-time performance of the algorithm. The results of simulation and experiment show the correctness and excellent estimation performance of the STMCKF algorithm proposed in this paper. The comparison of Table 2 and RMSE shows that the accuracy of STMCKF is much higher than that of EKF and CKF while the time consumption of STMCKF is only 31.91% longer than that of EKF and 40.62% shorter than that of CKF. For the sudden change of robot motion state, STMCKF can also make accurate judgment, effective suppression and strong tracking. To sum up, STMCKF is very suitable for the quadruped robot system whose motion state changes constantly. Using the proposed STMCKF algorithm, the state estimation accuracy of quadruped robots can be effectively improved without increasing the research and development (R&D) cost. Moreover, the proposed algorithm has good robustness and real-time performance, making the sensor on the robot achieve the maximum use benefit. Meanwhile, since there is no limitation to the number of feet, SMSRCKF is suitable for all legged robots, which has great application value for improving the motion performance of legged robot and for enhancing the accuracy of navigation and positioning.

## Figures and Tables

**Table 1:** The D-H parameter table of right front leg of the quadruped robot.

Joint i	Connecting Rod Length $l_{i}$ (mm)	Torsional Angle $\alpha_{i}$ (°)	Connecting Rod Distance $d_{i}$ (mm)	Connecting Rod Angle $\theta_{i}$ (°)
1	100	90	0	$\theta_{1}$
2	315	0	0	$\theta_{2}$
3	335	0	0	$\theta_{3}$
4	340	0	0	$\theta_{4}$

**Table 2:** Running time and increased percentage of three filters running 100,000 times.

Algorithm	EKF	CKF	STMCKF
Running time (s)	1.288	2.861	1.699
Increased (%)	0	122.13	31.91

## Appendix A

The optimal state estimation based on Kalman filtering derivation has the following forms:

(A1) $${\hat{x}}_{k}={\hat{x}}_{k/k-1}+E\left({\tilde{x}}_{k/k-1}{\tilde{z}}_{k/k-1}\right){\left(E\left({\tilde{z}}_{k/k-1}{\tilde{z}}_{k/k-1}^{T}\right)\right)}^{-1}{\tilde{z}}_{k/k-1}$$

where,

(A2) $${\tilde{z}}_{k/k-1}=z_{k}-\sum_{i=0}^{2n+2}\omega_{i}h\left(\delta_{i}\right)$$

(A3) $$E\left({\tilde{z}}_{k/k-1}{\tilde{z}}_{k/k-1}^{T}\right)=P_{zz,k/k-1}=H_{k}P_{k/k-1}H_{k}^{T}+R_{k}$$

(A4) $$E\left({\tilde{x}}_{k/k-1}{\tilde{z}}_{k/k-1}^{T}\right)=P_{xz,k/k-1}=P_{k/k-1}H_{k}^{T}$$

Introduce the fading factor according to the traditional method and compensate the innovation, then,

(A5) $$P_{k/k-1}^{*}=\lambda_{k}F_{k-1}P_{k-1}F_{k-1}^{T}+Q_{k-1}$$

(A6) $$P_{zz,k/k-1}^{*}=H_{k}P_{k/k-1}^{*}H_{k}^{T}+R_{k}$$

(A7) $$P_{xz,k/k-1}^{*}=P_{k/k-1}^{*}H_{k}^{T}$$

(A8) $${\tilde{z}}_{k/k-1}^{*}=z_{k}-\sum_{i=0}^{2n+2}\omega_{i}h\left(\delta_{i}^{*}\right)$$

$\delta_{j}^{*}$ is the volume point generated by $P_{k+1/k}^{*}$ after the introduction of the fading factor.

(A9) $${\hat{x}}_{k}^{*}={\hat{x}}_{k/k-1}+P_{xz,k/k-1}^{*}P_{zz,k/k-1}^{*-1\;}{\tilde{z}}_{k/k-1}^{*}$$

The two formulas subtract each other, then,

(A10) $${\hat{x}}_{k}-{\hat{x}}_{k}^{*}=P_{xz,k/k-1}^{*}P_{zz,k/k-1}^{*-1\;}\left({\tilde{z}}_{k/k-1}-{\tilde{z}}_{k/k-1}^{*}\right)=P_{xz,k/k-1}^{*}P_{zz,k/k-1}^{*-1\;}\sum_{i=0}^{2n+2}\omega_{i}\left(h\left(\delta_{i}\right)-h\left(\delta_{i}^{*}\right)\right)$$

Thus, when strong tracking is valid, that is, $\lambda_{k}>1$, then $P_{k/k-1}\neq P_{k/k-1}^{*}$, then $\delta_{i}\neq \delta_{i}^{*}$. That is, the state estimation results are biased. The performance index formula (3) is thus not met. It can be seen that compensation for one-step prediction of observation is unnecessary.

When the fading factor is introduced according to the traditional method, but the innovation is not compensated, there is,

(A11) $${\hat{x}}_{k}^{*}={\hat{x}}_{k/k-1}+P_{xz,k/k-1}^{*}P_{zz,k/k-1}^{*-1\;}{\tilde{z}}_{k/k-1}$$

Then, the performance index formula (3) is met, but if strong tracking is effective at this time, which means $\lambda_{k}>1$, there is still ${\tilde{z}}_{k/k-1}\neq {\tilde{z}}_{k/k-1}^{*}$.

Then, whether performance index formula (4) is met cannot be guaranteed. That is, the innovation sequence cannot be guaranteed to be orthogonal.

## Appendix B

First, the system state equation and observation equation are expanded by Taylor series at $x_{k-1}={\hat{x}}_{k-1}$ and $x_{k}={\hat{x}}_{k/k-1}$, then:

(A12) $$x_{k}\approx f_{k-1}\left({\hat{x}}_{k-1}\right)+F_{k-1}\left(x_{k-1}-{\hat{x}}_{k-1}\right)+w_{k-1}$$

(A13) $$z_{k}\approx h_{k}\left({\hat{x}}_{k/k-1}\right)+H_{k}\left(x_{k}-{\hat{x}}_{k/k-1}\right)+v_{k}$$

where,

$H_{k}=\frac{\partial h_{k}\left(x_{k}\right)}{\partial x_{k}}|{}_{x_{k}={\hat{x}}_{k/k-1}}$, $F_{k-1}=\frac{\partial f_{k-1}\left(x_{k-1}\right)}{\partial x_{k-1}}|{}_{x_{k-1}={\hat{x}}_{k-1}}$.

The innovation ${\tilde{z}}_{k}$ at the moment k is:

(A14) $$\begin{array}{l} {\tilde{z}}_{k}=z_{k}-{\hat{z}}_{k}=h_{k}\left(x_{k}\right)+v_{k}-\sum_{i=0}^{2n+2}\omega_{i}h_{k}\left(x_{k,i}\right)\\ \approx h_{k}\left({\hat{x}}_{k/k-1}\right)+H_{k/k-1}\left(x_{k}-{\hat{x}}_{k/k-1}\right)+v_{k}-\sum_{i=0}^{2n+2}\omega_{i}h_{k}\left(x_{k,i}\right) \end{array}$$

${\hat{z}}_{k}$ can be expanded into the following form:

(A15) $${\hat{z}}_{k}=\sum_{i=0}^{2n+2}\omega_{i}h_{k}\left(x_{k,i}^{l}\right)=E\left[h_{k}\left(x_{k}\right)\right]=h_{k}\left({\hat{x}}_{k/k-1}\right)+\left(\frac{\nabla P_{k/k-1}\nabla^{T}}{2}\right)h_{k}\left({\hat{x}}_{k/k-1}\right)$$

where ∇ is a complete differential operator. Substitute formula (A13) and formula (A15) into formula (A14), then,

(A16) $${\tilde{z}}_{k}=H_{k}\left(x_{k}-{\hat{x}}_{k/k-1}\right)+v_{k}-\left(\frac{\nabla P_{k/k-1}\nabla^{T}}{2}\right)h_{k}\left({\hat{x}}_{k/k-1}\right)$$

Similar to formula (A15), ${\hat{x}}_{k/k-1}$ can be expanded into the following form:

(A17) $${\hat{x}}_{k/k-1}=\sum_{i=0}^{2n+2}\omega_{i}\chi_{k/k-1,i}=E\left[f_{k-1}\left(x_{k-1}\right)\right]=f_{k-1}\left({\hat{x}}_{k-1}\right)+\left(\frac{\nabla P_{k-1}\nabla^{T}}{2}\right)f_{k-1}\left({\hat{x}}_{k-1}\right)$$

Calculate $x_{k}-{\hat{x}}_{k/k-1}$ based on formula (A12) and formula (A17), then,

(A18) $$\begin{array}{l} x_{k}-{\hat{x}}_{k/k-1}=f_{k-1}\left(x_{k-1}\right)+w_{k-1}-\sum_{i=0}^{2n+2}\omega_{i}f_{k-1}\left(x_{k-1,i}\right)\\ \approx f_{k-1}\left({\hat{x}}_{k-1}\right)+F_{k-1}\left(x_{k-1}-{\hat{x}}_{k-1}\right)+w_{k-1}-f_{k-1}\left({\hat{x}}_{k-1}\right)-\left(\frac{\nabla P_{k-1}\nabla^{T}}{2}\right)f_{k-1}\left({\hat{x}}_{k-1}\right)\\ =F_{k-1}\left(x_{k-1}-{\hat{x}}_{k-1}\right)+w_{k-1}-\left(\frac{\nabla P_{k-1}\nabla^{T}}{2}\right)f_{k-1}\left(x_{k-1}\right) \end{array}$$

Substitute formula (A18) into formula (A16), then,

(A19) $$\begin{array}{l} {\tilde{z}}_{k}=H_{k}F_{k-1}\left(x_{k-1}-{\hat{x}}_{k-1}\right)+H_{k}w_{k-1}-H_{k}\left(\frac{\nabla P_{k-1}\nabla^{T}}{2}\right)f_{k-1}\left({\hat{x}}_{k-1}\right)\\ +v_{k}-\left(\frac{\nabla P_{k/k-1}\nabla^{T}}{2}\right)h_{k}\left({\hat{x}}_{k/k-1}\right) \end{array}$$

$x_{k-1}$ can be expanded by Taylor series into:

(A20) $$x_{k-1}\approx f_{k-2}\left({\hat{x}}_{k-2}\right)+F_{k-2}\left(x_{k-2}-{\hat{x}}_{k-2}\right)+w_{k-2}$$

(A21) $${\hat{x}}_{k-1}={\hat{x}}_{k-1/k-2}+K_{k-1}{\tilde{z}}_{k-1}\approx f_{k-2}\left({\hat{x}}_{k-2}\right)+\left(\frac{\nabla P_{k-2}\nabla^{T}}{2}\right)f_{k-2}\left({\hat{x}}_{k-2}\right)+K_{k-1}{\tilde{z}}_{k-1}$$

(A22) $$\begin{array}{l} x_{k-1}-{\hat{x}}_{k-1}=F_{k-2}\left(x_{k-2}-{\hat{x}}_{k-2}\right)+w_{k-2}-\left(\frac{\nabla P_{k-2}\nabla^{T}}{2}\right)f_{k-2}\left({\hat{x}}_{k-2}\right)-K_{k-1}{\tilde{z}}_{k-1}\\ =\left(F_{k-2}-K_{k-1}H_{k-1}F_{k-2}\right)\left(x_{k-2}-{\hat{x}}_{k-2}\right)+\left(I-K_{k-1}H_{k-1}\right)w_{k-2}-K_{k-1}v_{k-1}\\ -\left(I-K_{k-1}H_{k-1}\right)\left(\frac{\nabla P_{k-2}\nabla^{T}}{2}\right)f_{k-2}\left({\hat{x}}_{k-2}\right)+K_{k-1}\left(\frac{\nabla P_{k-1/k-2}\nabla^{T}}{2}\right)h_{k-1}\left({\hat{x}}_{k-1/k-2}\right) \end{array}$$

Substitute formula (A23) into formula (A19), then the covariance at any previous time is:

(A23) $$E\left[\left(\frac{\nabla P_{k-2}\nabla^{T}}{2}\right)f_{k-2}\left({\hat{x}}_{k-2}\right){\tilde{z}}_{k-j}^{T}\right]=\left(\frac{\nabla P_{k-2}\nabla^{T}}{2}\right)f_{k-2}\left({\hat{x}}_{k-2}\right)E\left({\tilde{z}}_{k-j}^{T}\right)=0$$

(A24) $$E\left[\left(\frac{\nabla P_{k-1/k-2}\nabla^{T}}{2}\right)h_{k-1}\left({\hat{x}}_{k-1}\right){\tilde{z}}_{k-j}^{T}\right]=\left(\frac{\nabla P_{k-1/k-2}\nabla^{T}}{2}\right)h_{k-1}\left({\hat{x}}_{k-1}\right)E\left({\tilde{z}}_{k-j}^{T}\right)=0$$

(A25) $$E\left[w_{k-2}{\tilde{z}}_{k-j}^{T}\right]=0$$

(A26) $$E\left[v_{k-2}{\tilde{z}}_{k-j}^{T}\right]=0$$

Then the covariance of the innovation sequence at any previous time k−j is:

(A27) $$\begin{array}{l} E\left[{\tilde{z}}_{k}{\tilde{z}}_{k-j}^{T}\right]=E\left[H_{k}F_{k-1}\left(F_{k-2}-K_{k-1}H_{k-1}F_{k-2}\right)\left(x_{k-2}-{\hat{x}}_{k-2}\right){\tilde{z}}_{k-j}^{T}\right]\\ =H_{k}F_{k-1}\left(F_{k-2}-K_{k-1}H_{k-1}F_{k-2}\right)E\left[\left(x_{k-2}-{\hat{x}}_{k-2}\right){\tilde{z}}_{k-j}^{T}\right] \end{array}$$

It can be seen that there is an iterative connection between $E\left[{\tilde{z}}_{k}{\tilde{z}}_{k-j}^{T}\right]$ and $\left(x_{k-2}-{\hat{x}}_{k-2}\right)$. According to formula (A22), the above steps are iterated, and it can be finally obtained that,

(A28) $$E\left[{\tilde{z}}_{k}{\tilde{z}}_{k-j}^{T}\right]=H_{k}F_{k-1}\left(F_{k-2}-K_{k-1}H_{k-1}F_{k-2}\right)\left(F_{k-j}-K_{k-j+1}H_{k-j+1}F_{k-j}\right)E\left(P_{k-j/k-j-1}H_{k-j}^{T}-K_{k-j}V_{k-j}\right)$$

(A29) $$V_{k-j}=E\left[{\tilde{z}}_{k-j}{\tilde{z}}_{k-j}^{T}\right]$$

According to the principle of strong tracking orthogonality, $E\left[{\tilde{z}}_{k+j}{\tilde{z}}_{k}^{T}\right]=0$, sufficient condition must be met:

(A30) $$P_{k/k-1}H_{k}^{T}-K_{k}V_{k}=0$$

Formula (A30) can be written as:

(A31) $$P_{xz,k/k-1}-K_{k}V_{k}=0$$

(A32) $$K_{k}\left(P_{zz,k/k-1}-V_{k}\right)=0$$

Since $K_{k}$ is a non-zero matrix, then,

(A33) $$P_{zz,k/k-1}-V_{k}=0$$

It can be seen that calculating $\lambda_{k}$ while maintaining $P_{zz,k/k-1}=V_{k}$ can ensure that the innovation sequence is orthogonal everywhere.

(A34) $$\lambda_{k}\sum_{i=0}^{2n+2}\omega_{i}\zeta_{k/k-1,i}\zeta_{k/k-1,i}^{T}-{\hat{z}}_{k/k-1}{\hat{z}}_{k/k-1}^{T}=V_{k}-R_{k}$$

(A35) $$N_{k}=V_{k}-R_{k}$$

(A36) $$M_{k}=\sum_{i=0}^{2n+2}\omega_{i}\zeta_{k/k-1,i}\zeta_{k/k-1,i}^{T}-{\hat{z}}_{k/k-1}{\hat{z}}_{k/k-1}^{T}$$

(A37) $$V_{k}=\{\begin{array}{c} {\tilde{z}}_{k}{\tilde{z}}_{k}^{T},k=0\\ \frac{\rho V_{k-1}+{\tilde{z}}_{k}{\tilde{z}}_{k}^{T}}{1+\rho },k\geq 1 \end{array}$$

Then the matrix of the fading factor is as follows:

(A38) $$\lambda_{k}^{i}=\{\begin{array}{c} \alpha_{i}c_{k},\alpha_{i}c_{k}>1\\ 1,\alpha_{i}c_{k}\leq 1 \end{array},c_{k}=\frac{tr[N_{k}]}{\sum_{i=1}^{n}\alpha_{i}M_{k}^{ii}}$$

where $\alpha_{i}\geq 1$, $\lambda_{k}^{i}$ is the element of the fading factor matrix; $M_{k}^{ii}$ is the element on the diagonal of the matrix $M_{k}$; and the $\alpha_{i}$ value is obtained from RBF neural network training, which can also be obtained from prior knowledge.
